# The Prognostic Impact of the Surgical Margin in Renal Cell Carcinoma Treated with Partial Nephrectomy: A Multi-Center Study

**DOI:** 10.3390/cancers16081449

**Published:** 2024-04-09

**Authors:** Karina Sif Søndergaard Mosholt, Mark Aagaard, Andreas Røder, Nessn Azawi

**Affiliations:** 1 Department of Urology, Rigshospitalet, Blegdamsvej 9, 2100 Copenhagen, Denmark; karina.sif.soendergaard.mosholt@regionh.dk (K.S.S.M.); andreas.roeder@regionh.dk (A.R.); 2 Department of Urology, Zealand University Hospital, Sygehsuvej 10, 4000 Roskilde, Denmark; 3 Institute of Clinical Medicine, University of Copenhagen, Blegdamsvej 3B, 2100 Copenhagen, Denmark

**Keywords:** nephron-sparing surgery, positive surgical margins, renal cell carcinoma, robotic-assisted surgery, cancer recurrence

## Abstract

**Simple Summary:**

Patients with small kidney cancers who undergo tumor resection, can experience incomplete tumor tissue removal. This is termed “positive surgical margin” (PSM) and is of interest in terms of the risk for cancer recurrence and survival outcomes. In this study, we investigated outcomes in 523 patients, of which 48 patients had PSM after tumor resection. We found positive margin rates to be lower in robot-assisted tumor resection compared to traditional methods (5.5% vs. 12.2%). Patients with a PSM had higher rates of recurrence during follow-up compared to patients with negative margins (23% vs. 9.3%), highlighting the impact of surgical approach in preventing PSM and thereby influencing the risk of cancer recurrence. However, the presence of PSM did not affect patient survival.

**Abstract:**

**Background:** Partial nephrectomy (PN) is the preferred treatment for small, localized kidney tumors. Incomplete resection resulting in positive surgical margins (PSM) can occur after PN. The impact of PSM on the risk of recurrence and survival outcomes is not fully understood. We aimed to explore the relationship between PSM, the risk of recurrence and impact on survival after PN in a large multicenter cohort from Denmark. **Methods:** This was a retrospective cohort study including patients who underwent PN for renal cell carcinoma (RCC) at three departments in Denmark between 2010 and 2016. Data including pathological features, surgical techniques, and patient follow-up was retrieved from electronic medical health records and national databases. We used a combination of descriptive statistics, comparative analysis (comparisons were carried out by Mann–Whitney Test, independent Student’s *t*-test, or Pearson’s chi-Square Test), univariate and multivariate logistic regression analyses, and survival analysis methods. **Results:** A total of 523 patients were included, of which 48 (9.1%) had a PSM. Recurrence was observed in 55 patients (10.5%). Median follow-up time was 75 months. We found a lower incidence of PSM with robot-assisted PN (*p* = 0.01) compared to open or laparoscopic PN. PSM was associated with a higher risk of recurrence compared to negative margins in univariate analysis, but not multivariate analysis. However, the study was underpowered to describe this association with other risk factors. Overall survival did not differ between patients with PSM and negative margins. **Conclusions:** Our study presents further evidence on the negative impact of PSM on recurrence after PN for RCC, highlighting the importance of achieving NSM, thus potentially improving clinical outcomes. A surgical approach was found to be the only predictive factor influencing the risk of PSMs, with a reduced risk observed with robot-assisted laparoscopy.

## 1. Introduction

Partial nephrectomy (PN) is increasingly advocated as the preferred treatment option for small localized renal cell carcinomas (RCCs). PN reduces the long-term risk of end-stage renal disease compared to radical nephrectomy, while maintaining an equivocal oncological outcome. With local surgical tumor resection comes a risk of histologically residual disease, known as positive surgical margin (PSM). The incidence of PSMs has been reported from 0 to 10.7% in previous PN cohorts [1], but the impact of PSMs on oncological outcomes remains elusive. Despite the histological presence of PSM, residual cells in the tumor bed may not be viable, e.g., due to surgical hemostasis, and thus never result in local or distant recurrence of the disease. The correlation between PSMs and overall survival (OS), cancer-specific survival (CSS), and recurrence-free survival (RFS) is an ongoing debate. Some research indicates that PSMs may escalate the risk of local recurrence and negatively impact oncological outcomes. Conversely, other studies suggest that survival rates are comparable regardless of the postoperative strategy, whether it involves active surveillance or surgical interventions [1,2,3,4].

The management of localized kidney tumors has progressively shifted towards nephron-sparing procedures, primarily to safeguard renal functions. This shift is underscored by findings that radical nephrectomy, in contrast to partial nephrectomy, is associated with diminished long-term survival due to an elevated risk of chronic kidney disease, which further escalates the likelihood of cardiovascular-related morbidity and mortality. Since 2010, nephron-sparing surgery (NSS) has been advocated as the standard approach where feasible, successfully balancing the dual objectives of preserving kidney function and ensuring satisfactory oncological outcomes [5].

Building upon these considerations, our study aims to investigate the relationship between PSMs and recurrence risk, alongside evaluating the influence of PSMs on survival outcomes in a patient cohort that underwent PN for RCC.

## 2. Method

### 2.1. Data Collection and Database Description

Patients were identified through the Danish Renal Cancer (DaRenCa) database, which is comprised of specific details reported on RCC patients from all Urology Departments in Denmark. The database integrates comprehensive data from multiple national registries: The Civil Registration System (CRS), the National Patient Registry (NPR), the Danish Pathology Registry (DPR), the Danish Cancer Registry (DCR), and the Cause of Death Register (DAR). Patients included in the cohort had undergone PN for the treatment of RCC between 2010 and 2016 in one of the three urological departments located in the east region of Denmark. The inclusion of patients in this specific time interval secured a relevant follow-up period of at least 5 years. Patients with bilateral tumors, lymph node involvement, and metastatic disease were excluded from the study.

To retrieve data from the database, approval through the Danish Data Agency is required.

### 2.2. Preoperative Patient Characteristics

Additional patient characteristics were obtained from reviewing medical records, including demographics, performance score (PS), American Society of Anesthesiologists (ASA) score, Charlson Comorbidity Index (CCI), and symptoms such as hematuria, pain, and weight loss. Smoking status and pertinent laboratory results were also recorded. PADUA nephrometry scores were reviewed from cross-sectional CT scans.

### 2.3. Tumor Characteristics and Surgical Aspects

Pathological characteristics were retrieved from pathology reports, and included tumor diameter, pTNM staging, histologic subtype, Fuhrmann nuclear grade classification, presence of necrosis, Leibovich score for clear cell RCCs, and surgical margin status. Tumor diameter, histological subtype, and nuclear grade was evaluated according to national guidelines. PSM was defined as the presence of malignant cells in the resection margin of the removed tumor, and NSM was verified if non-malignant cells were observed in the resection margin. Surgical details retrieved from medical records were the utilized surgical approach (open, laparoscopic or robot-assisted laparoscopic surgery), whether clamping was performed, and resection techniques (enucleation (tissue-sparing) vs. resection (tumor removal with inclusion of tumor and normal kidney tissue)).

### 2.4. Follow-Up Protocol and Recurrence Assessment

All participating institutions adhered to the national DaRenCa guidelines for postoperative follow-up, entailing a thoraco-abdominal CT-scan 6 months post-surgery, regardless of margin status. Patients with PSM endured a closer follow-up regimen, with CT-scans performed every 6 months for three years, followed by annual scans. For patients with NSM, follow-up CT-scans were situated 6 and 12 months postoperatively, followed by annual scans during the remaining study period.

Recurrence was identified through radiologic evaluations and, where applicable, pathologic analysis of recurrent tumors. Local recurrences were defined as those occurring at the surgical field, while recurrences at other sites could consist of multiple site recurrences or metastases. This study’s primary focus was to assess oncological outcomes and, secondarily, to evaluate potential risk factors for PSM and recurrence.

We used a combination of descriptive statistics, comparative analysis (comparisons were carried out by Mann–Whitney Test, independent Student’s *t*-test, or Pearson’s chi-Square Test). The estimation of the model’s baseline hazard was established using Breslow’s method, a standard approach in survival analysis.

### 2.5. Ethical Approvals

The study was approved be Danish Data Agency and the Danish Patient Safety Authority (3-3013-2902/1).

## 3. Results

### 3.1. Patient Demographics and Baseline Characteristics

A total of 523 patients underwent PN. A PSM was identified in 48 (9.2%) patients, while recurrences were observed in 55 (10.5%). The median follow-up time was 75 months (interquartile range, IQR: 58–91).

Patient characteristics are presented in Table 1. We did not find differences in the distribution of preoperative characteristics when stratifying for surgical margin status, but PSM was more incident in patients with higher-stage tumors, and less incident in patients who underwent robot-assisted PN. Patient allocation between the PSM and NSM groups was generally even, aside from notable disparities in surgical methods, recurrence frequencies, and recurrence sites as detailed in Table 1. There was a higher occurrence of T2 and T3 stage tumors in the PSM group, with both stages at 8.5%, in contrast to the NSM group, which had 4.7% for T2 and 2.5% for T3 tumors. Nonetheless, the mean tumor size was consistent between the two cohorts. Additionally, the distribution of tumor nuclear grades, classified as low (Fuhrmann 1–2) and high (Fuhrmann 3–4), showed no significant difference when comparing the PSM and NSM groups.

### 3.2. Positive Surgical Margin

The surgical approach emerged as a critical factor influencing the risk of PSM. Robotic-assisted procedures were associated with a lower risk of PSM (5.5%) compared to laparoscopic and open surgeries (12.2% each, *p* = 0.01). The analysis did not show significant differences concerning clamping (yes/no) and resection techniques (enucleation/resection) between the PSM and NSM groups (Table 2).

### 3.3. Recurrences

In the univariate analysis, several factors were found to have a significant relationship with recurrence. PSM showed a notable association (HR 2.27; 95% CI 1.17 to 4.40; *p* = 0.02), indicating a more than twofold increase in hazard compared to NSM. Similarly, the Fuhrman grade was linked to recurrence (HR 1.80; 95% CI: 1.21–2.68; *p* < 0.005). Tumor size also demonstrated a significant correlation with recurrences (HR 1.02; 95% CI: 1.01–1.03; *p* < 0.005). Furthermore, the Leibovich score was significantly associated with recurrences (HR 1.48; 95% CI: 1.20–1.82; *p* < 0.005), reflecting a meaningful increase in hazard with a higher Leibovich score. Conversely, age, PADUA score, pathological tumor stage, presence of necrosis, and resection technique (excision or enucleation) did not exhibit statistically significant effects on the recurrences time (Table 3).

The multivariate analysis identified only two significant predictors of recurrence. The presence of a PSM was associated with a more than twofold increase in the risk of the recurrence (HR 2.05; 95% CI: 1.04–4.04; *p* = 0.04). Furthermore, the Leibovich score was also a significant prognostic factor. For each unit increase in the Leibovich score, there was a 30% increase in the hazard (HR 1.30; 95% CI: not provided; *p* < 0.005). Conversely, recurrence was not significantly associated with gender, age, and surgical. The model’s concordance index was 0.69 (Table 4).

### 3.4. Survival Outcomes

**Figure 1:**
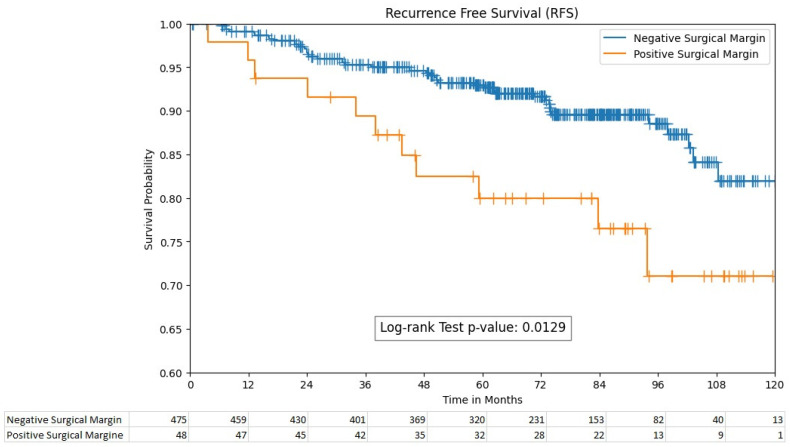
Recurrence-free survival by surgical margin status.

The 5-year recurrence-free survival rates were significantly different between the PSM and NSM groups, with rates of 80% (95% CI; 64.9–89.1) and 93% (95% CI; 90.1–95.1), respectively ((*p* = 0.013), Figure 1). However, the 5-year overall survival rates between these groups were non-significant, with 87% (95% CI; 84–90) for the PSM group and 91% (95% CI; 78.7–96.7) for the NSM group (*p* = 0.055).

## 4. Discussion

The findings from our study underscore the significant association between the surgical approach and the risk of PSM in partial nephrectomy procedures. The data reveal a compelling trend towards robotic-assisted surgeries being associated with a notably lower risk of PSM at 5.5%, compared to their laparoscopic and open surgery counterparts, each presenting a PSM risk of 12.2%. This differential is statistically significant (*p* = 0.01). In Denmark, robotic-assisted PN was introduced in 2008, and was a relatively novice approach in the beginning of our cohort period. Most surgeons performing PN in this cohort were experienced kidney surgeons, but not all were experienced console surgeons. Despite this, in our cohort, PSMs were less likely to occur with robotic assisted surgery, suggesting that the learning curve for the experienced kidney surgeon is steep, and the precision and enhanced dexterity offered by robotic assistance may contribute to more accurate tumor resection and consequently better surgical outcomes. This could be particularly beneficial in complex renal masses where the preservation of healthy tissue is paramount for maintaining renal function, while ensuring complete tumor excision [6,7,8].

The results of our study provide a comprehensive analysis of factors influencing recurrence and survival outcomes in patients undergoing partial nephrectomy for RCC. In the univariate analysis, PSM, Fuhrman grade, tumor size, and Leibovich score emerged as significant predictors of recurrence. Notably, PSM was associated with a more than twofold increase in the risk of recurrence, which is consistent with the literature that identifies the surgical margin status as a critical prognostic indicator [9,10].

The Leibovich score, tumor size, and Fuhrmann grade, among other factors, were also significantly associated with recurrence in our study. This is in agreement with previous studies that have validated the Leibovich scoring system as a reliable predictor of recurrence in RCC [11,12,13].

Regarding survival outcomes, our study demonstrated a significant difference in 5-year recurrence-free survival rates between the PSM and NSM groups, with the NSM group showing superior outcomes. This finding is in line with the established notion that PSM is a negative prognostic factor for recurrence-free survival. However, the 5-year overall survival rates did not significantly differ between the two groups. This may suggest that while PSM is a strong predictor of recurrence, it may not independently predict overall survival, a phenomenon that has been observed in other studies [1,14,15,16,17,18,19]. This could be attributed to the indolent nature of some RCC cases, where recurrence does not immediately translate to a survival disadvantage.

Previous studies have reported a high risk of recurrence in patients with PSM following partial nephrectomy [20,21], which is in agreement with our findings. Khalifeh et al. [6] reported a hazard ratio of 18 for recurrence at a 17.3-month follow-up, while Takagi et al. [17] found a median time to recurrence after PN of 9 months. However, our cohort had a follow-up time of at least 5 years, which is considered sufficient to predict recurrence.

The decision to use off-clamp or clamping techniques during PN can significantly impact patient outcomes [22,23,24]. Clamping the renal artery reduces blood loss, but can cause ischemia to the kidney, while off-clamp techniques avoid ischemia but may lead to more bleeding. We found no significant differences in PSM occurrence between these two techniques, which is a valuable insight. Further analysis could explore how these techniques interact with tumor complexity and surgeon experience. For instance, more experienced surgeons might be more adept at managing complex tumors without clamping, thus preserving renal function without compromising surgical margins.

Interestingly, our study did not find a significant association between recurrence and other factors such as age, PADUA score, tumor stage, presence of necrosis, and resection technique. This contrasts with some studies that have suggested these factors may influence recurrence risk [11,25]. The lack of statistical significance in our study could be due to sample size or the distribution of these factors within our cohort.

The management of positive surgical margins (PSM) following partial nephrectomy for renal cell carcinoma (RCC) is a subject of ongoing debate [21,26]. There is no clear consensus on the optimal approach. While some studies have suggested that repeat resection of the tumor bed may be necessary, other studies have found comparable survival rates between patients with PSM and NSM, regardless of postoperative management regimes including active surveillance versus repeat resection of the tumor bed [3,4,27].

Data on treatment strategies upon recurrence are not included in this study. In future, it will be interesting to observe whether adjuvant treatment in the era of targeted systemic therapies will play a role in managing PSM. Furthermore, a comparison of survival data between patients with recurrence after PSMs in this cohort and a more present cohort could uncover whether novice oncological treatments will present a positive influence on survival outcomes. Furthermore, extending the cohort to include all patients who have undergone PN in a specific time frame in all regions of Denmark is relevant to further evaluate the significance of PSM.

Study limitations potentially affecting data interpretation include its retrospective design, which could mediate selection bias. Furthermore, there was missing data for some patients, including smoking status, performance score, information on resection technique, clamping, and Fuhrmann nuclear grade, which could potentially influence the interpretation of data Additionally, the study did not include information on the treatment for recurrence, which could influence outcomes.

## 5. Conclusions

Our study presents further evidence on the negative impact of PSM on recurrence after PN for RCC, highlighting the importance of achieving NSM, thus potentially improving clinical outcomes. Surgical approach was found to be the only predictive factor influencing the risk of PSM, with a reduced risk observed with robotic-assisted laparoscopy. The optimal strategy for managing PSM remains unclear, and further research is needed to determine the best approach for patients with PSM, especially those with high-risk tumors. The lack of impact of PSM on overall survival warrants further investigation, potentially exploring the role of adjuvant therapies and the biological behavior of different RCC subtypes.

## Figures and Tables

**Table 1:** Baseline and perioperative demographics for patients with microscopic PSM/NSM following PN for RCC.

Characteristic	PSM (*n* = ) *N* (%)	NSM (*n* = ) *N* (%)	*p*-Value
**Age (median, IQR)**	61.2 (54.4–69.1)	64.1 (55.3–69.7)	0.57
**Gender**			0.24
Male	36 (75%)	317 (66.7%)	
Female	12 (25%)	158 (33.3%)	
**Performance score**			0.93
0	38	355	
1	8	77	
2	2	27	
3	0	2	
**Body Max Index (median, IQR)**	26.5 (23.1–31.6)	26.8 (24.1–30.1)	0.84
**Charlson score index**			0.37
0	132	9	
1	169	23	
2	108	12	
3	45	4	
4	16	0	
5	5	0	
**Hypertension**	28 (58.3%)	239 (51.2%)	0.35
**Symptoms**	13 (27.7%)	167 (36.9%)	0.21
**PADUA score (median, IQR)**	9 (7–11)	8 (7–10)	0.26
**Smoking**	*N* = 45	*N* = 442	0.24
Current	18 (40%)	129 (29.2%)	
Previous	11 (24.4%)	153 (34.6%)	
Never	16 (35.6%)	160 (36.2%)	
**Tumor localization**			0.63
Right kidney	23 (47.9%)	236 (50.1%)	
Left kidney	23 (47.9%)	224 (47.6%)	
Bilateral	2 (4.2%)	11 (2.3%)	
**Tumor size (mm) (median, IQR)**	30 (23–40)	30 (21–41)	0.35
**Surgical technique**			
Laparoscopic	16 (34%)	110 (23.8)	
Open	18 (38.3%)	130 (28.1%)	
Robotic	13 (27.7%)	222 (48.1%)	0.03
**Clamping technique**	*N* = 47	*N* = 433	0.47
Yes	39 (86.7%)	390 (90.1%)	
No	6 (13.3%)	43 (9.9%)	
**Resection technique**	*N* = 45	*N* = 439	0.11
Excision	42 (93.3%)	428 (97.5%)	
Enucleation	3 (6.7%)	11 (2.5%)	
**Histology**	*N* = 46	*N* = 467	0.49
Clear cell	27 (58.7%)	323 (69.2%)	
Non-clear cell	19 (41.3%)	144 (30.8%)	
**T-category**			0.06
T1a	32 (68.1%)	344 (72.9%)	
T1b	7 (14.9%)	94 (19.9%)	
T2	4 (8.5%)	22 (4.7%)	
T3a/b	4 (8.5%)	12 (2.5%)	
**Nuclear Grade**	*N* = 42	*N* = 459	0.29
Fuhrmann 1	5 (11.9%)	111 (24.2%)	
Fuhrmann 2	29 (69%)	272 (59.3%)	
Fuhrmann 3 and 4	8 (19.1%)	76 (16.5%)	
**Recurrences**			
**No**	37 (77%)	431 (91%)	0.01
**Yes**	11 (23%)	44 (9%)	
**Site of recurrence**	11	44	0.007
Local	9	24	
Lunge +	1	9	
Bone +	0	3	
Other +	1	8	
**Died**	6	103	0.14

**Table 2:** Potential risk factors for positive resection margin compared to negative resection margin in partial nephrectomy.

Risk Factors	Categories	Number (%)	*p*-Value
Surgical approach	Laparoscopic	16 (12.7%)	0.01
	Open	18 (12.2%)	
	Robot	13 (5.5%)	
pT-category	T1	1 (33.3%)	0.45
	T1a	31 (8.3%)	
	T1b	7 (6.9%)	
	T2a	4 (20%)	
	T3a	3 (23%)	
	T3b	1 (50%)	
Resection technique	Enucleation	3 (21.4%)	0.28
	Excision	42 (8.9%)	
Histologic subtype	clear cell adenocarcinoma	27 (7.7%)	0.21
	chromofobe renal cell carcinoma	7 (14.9%)	
	papillary adenocarcinoma type 1	8 (14.5%)	
	papillary adenocarcinoma type 2	4 (8.9%)	
Leibovich score	0	21 (7.6%)	0.19
	1	8 (10.1%)	
	2	1 (1.5%)	
	3	4 (10.8%)	
	4	7 (21.2%)	
	5	3 (23.1%)	
	6	0 (0)	
	7	0 (0)	
Tumor size	<4 cm	36 (9.3%)	0.91
	4–7 cm	9 (8.3%)	
	>7 cm	3 (10.7%)	
Ischemia technique	Clamping	39 (9.1%)	0.52
	Off clamping	6 (12.2%)	

**Table 3:** Univariate analysis of potential predictors of recurrence rate.

Variable	Hazard Ratio	95% CI	*p*-Value
Resection technique	0.71	0.17 to 2.93	0.64
Necrosis	1.58	0.89 to 2.79	0.12
T-category	1.13	0.64 to 2.02	0.67
Fuhrman Grade	1.80	1.21 to 2.68	<0.005
PADUA score	1.12	0.97 to 1.30	0.13
Tumor size	1.02	1.01 to 1.03	<0.005
Leibovich score	1.48	1.20 to 1.82	<0.005
Age	1.24	0.93 to 1.65	0.14
Positive surgical margin	2.27	1.17 to 4.40	0.02

**Table 4:** Multivariate analysis of potential predictors of recurrence rate.

Variable	Coefficient	Hazard Ratio	95% CI	*p*-Value
PSM	0.72	2.05	0.04 to 1.40	0.04
Gender	0.31	1.37	−0.32 to 0.94	0.33
Leibovich score	0.26	1.3	0.11 to 0.42	<0.005
Age	0.02	1.02	−0.01 to 0.05	0.16
Surgical technique: Open	−0.2	0.82	−0.92 to 0.51	0.57
Surgical technique: Robot	−0.04	0.96	−0.71 to 0.63	0.91

## Data Availability

The data presented in this study is available in this article.
